# A Diagnostic Pitfall: Hidden Hazards of Lead Neuropathy by Co‐Existence of Hemoglobinopathy and Cervical Radiculomyelopathy

**DOI:** 10.1002/ccr3.70858

**Published:** 2025-09-05

**Authors:** Su Mon Thwe, Htoo Myat Myat Aung, Zin Nwe Win, Ohnmar Ohnmar

**Affiliations:** ^1^ Freelance Academic Tutor and Research Writer Yangon Myanmar; ^2^ Asia Royal Hospital and Yangon General Hospital Yangon Myanmar; ^3^ Yangon General Hospital Yangon Myanmar

**Keywords:** anemia, lead neuropathy, Myanmar, wrist drop

## Abstract

A 52‐year‐old Myanmar man presented with bilateral progressive painless asymmetrical wrist and finger drop in 1 year without any sensory and sphincter problems. He has hypochromic microcytic anemia diagnosed as Hemoglobin E disease before. However, a serial full blood count revealed thrombocytopenia and a drop in hemoglobin disproportionate to HbE disease. Neurophysiology testing indicated axonal motor polyneuropathy affecting the distal C7 and C8 muscles. His MRI showed spinal cord myelopathy and neural foraminal narrowing at C5–C6 and C6–C7, but MRI findings could not explain both his C8 weakness and electromyography findings of C8 radiculopathy. However, he was initially managed as Hemoglobin E disease and spondylotic cervical radiculomyelopathy. The patient has been operating a car workshop for over a decade, without any apparent history of direct exposure to lead‐contaminated batteries or lead‐based paint. However, due to suspicion of lead exposure raised by his family doctor, blood lead level testing was initiated. A more detailed occupational history revealed that the patient collected discarded air‐conditioning units in his workshop for recycling purposes and stored them in poorly ventilated spaces. Handling these items likely exposed him to a significant amount of lead dust and fumes. Finally, he was diagnosed with lead neuropathy, and after appropriate treatment, his muscle strength and blood counts returned to normal.

## Graphical abstract

Lead source: patient's workshop with repurposed old AC units and dynamos.

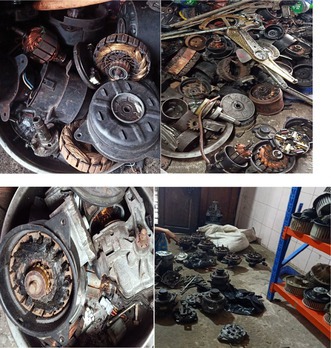

**Summary**

- Clinicians should maintain a high index of suspicion for lead neuropathy, especially in patients presenting with unexplained motor deficits and potential occupational exposure, particularly in settings with limited regulatory oversight.

## 1 Introduction

Lead is recognized as a neurotoxic metal with widespread historical use. Lead exposure primarily stems from industrial activities such as smelting and environmental remnants from contaminated soils, water, leaded gasoline residues, and aging infrastructure like lead‐based paints. These sources significantly contribute to lead's toxic effects on human health [1, 2]. Many developed and wealthy countries have implemented effective regulations and restrictions, significantly reducing lead exposure levels in recent years. However, developing countries still face risks from mining wastes and improper disposal and recycling of lead‐contaminated toys and electronics [3]. Little is known about environmental lead levels in Myanmar, and research has only been conducted on blood levels in Myeik, located in the extreme South of Myanmar [4]. Here, we report a case of lead neuropathy that was initially misdiagnosed as more commonly known conditions, such as hemoglobinopathy and spondylotic radiculopathy.

## 2 Case Presentation

### 2.1 Clinical Findings

A 52‐year‐old man, who operates a car workshop, presented with progressive bilateral wrist and finger drop, with the right side being affected for 1 year and the left side for 3 months. He reported no leg weakness, sensory deficits or bladder symptoms, cervical radicular pain, or any other pain. There was no history of trauma. He has no family history of neuromuscular weakness, and his medical history was notable only for anemia due to Hemoglobin E disease. On examination, he was fully alert (GCS 15), with normal cranial nerve function and cognition, and no signs of meningeal irritation. He exhibited bilateral wrist and finger drop, with 3/5 strength in bilateral wrist and finger extension, while all other muscle groups showed full strength (5/5). Tendon reflexes and sensory examination were normal, and he had bilateral flexor plantar responses. Other systemic examinations were normal apart from pallor. Our differential diagnoses included bilateral radial neuropathy, C7/C8 radiculopathy, multifocal motor neuropathy with conduction block (MMNCB), and chronic inflammatory demyelinating polyneuropathy (CIDP).

### 2.2 Diagnostic Focus and Management

**FIGURE 1:**
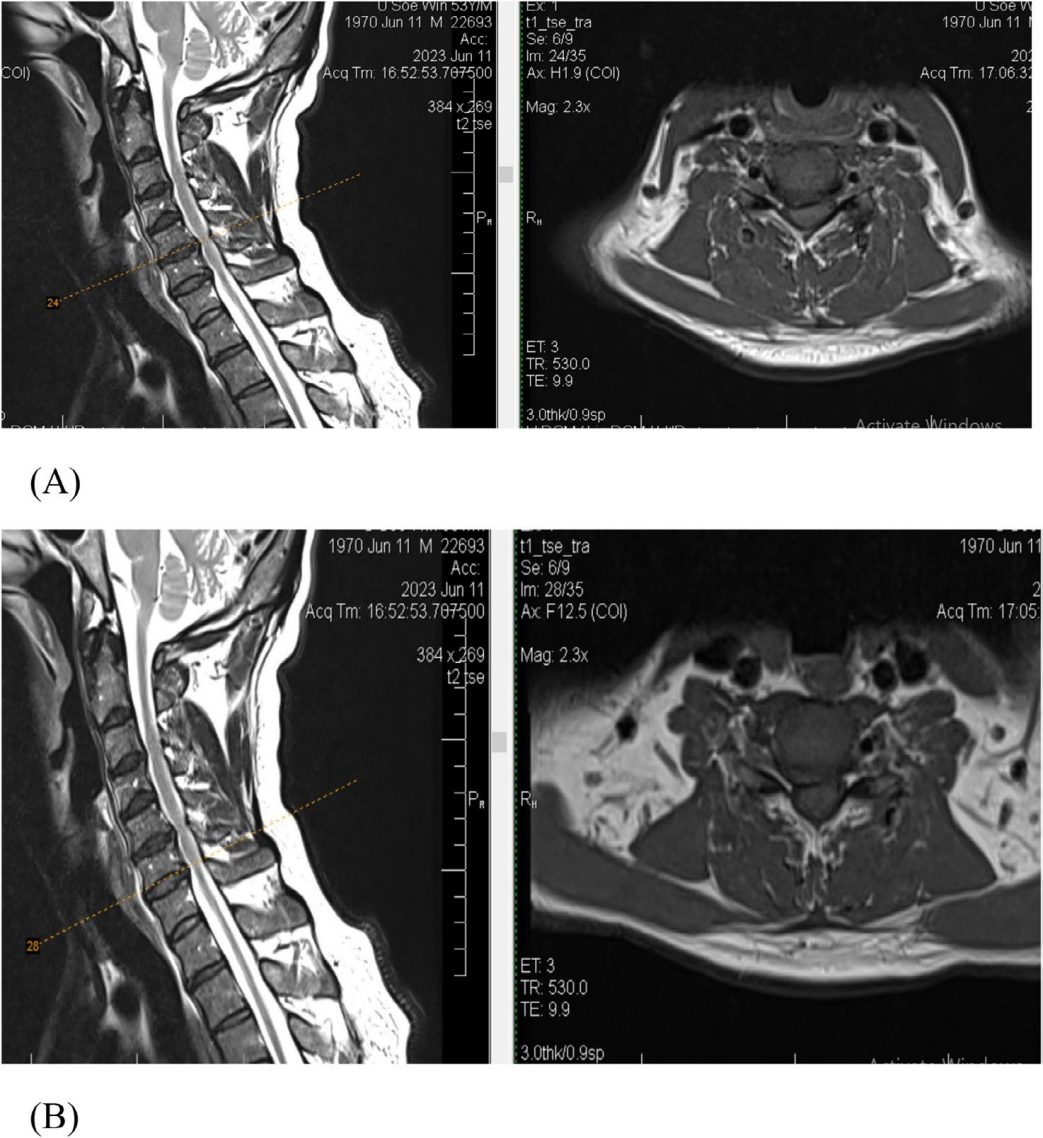
MRI images of the cervical spine demonstrating multilevel degenerative changes, most pronounced at the C5–C6 (A) and C6–C7 (B) levels. Notable findings include diffuse disc bulging, marginal osteophytes, ligamentum flavum thickening, causing spinal canal narrowing and moderate bilateral foraminal narrowing, and spinal cord T2 hyperintensity, consistent with cervical spondylotic radiculomyelopathy.

Upon investigation, ECG and chest X‐ray were unremarkable. An abdominal ultrasound revealed mild bilateral nephropathy. Blood tests showed hemoglobin (Hb) 7.9 g/dL (reference: 13.5–17.5 g/dL for males), mean corpuscular volume (MCV) 73 fL (80–100 fL), white blood cell count (WBC) 9.5 × 10^9^/L (4.0–11.0 × 10^9^/L), platelet count 94 × 10^9^/L (150–450 × 10^9^/L), and blood film revealed anisopoikilocytosis with hypochromia, microcytes, elliptical cells, fragmented cells, target cells, tear drop cells, relative neutrophilia with a left shift (2% band forms), lymphopenia, eosinophilia, and reduced platelet count. Hemoglobin electrophoresis confirmed heterozygous Hemoglobin E disease. Iron studies indicated iron (Fe) 12.72 μmol/L (5.83–34.5 μmol/L), total iron binding capacity (TIBC) 43.1 μmol/L (44.8–73.4 μmol/L), transferrin saturation 29.53% (20%–50%), and ferritin 589.1 μg/L (30–400 μg/L). Additional labs showed erythrocyte sedimentation rate (ESR) of 7 mm/hr (0–20 mm/hr), C‐reactive protein (CRP) 44.74 mg/L (< 5 mg/L), creatinine 145 μmol/L (60–110 μmol/L), urea 11.3 mmol/L (2.5–7.1 mmol/L), sodium 133 mmol/L (135–145 mmol/L), potassium 4.2 mmol/L (3.5–5.1 mmol/L), chloride 97 mmol/L (98–107 mmol/L), bicarbonate 24 mmol/L (22–29 mmol/L), and HbA1c 6% (4%–5.6%). Thyroid function, liver function tests, vitamin D, and urine analysis were all within normal limits. Screenings for HIV, HBV, HCV, and syphilis were negative. Motor and sensory nerve conduction studies were normal, without evidence of demyelination or conduction block. However, electromyography (EMG) indicated active denervation (increased spontaneous activities) and chronic reinnervation in muscles innervated by C7 and C8 (pronator teres, triceps, extensor indices, and first dorsal interossei), suggestive of C7, C8 radiculopathy. Tests for ENA, ANCA, and anti‐ganglioside antibodies were negative. MRI of the cervical spine showed degenerative changes with multilevel involvement, most severe at C5–C6 and C6–C7, along with spinal cord myelopathy (Figure 1).

He was diagnosed with Hemoglobin E disease and spondylotic cervical radiculomyelopathy. He was referred to a spine surgeon, and physiotherapy and rehabilitation were initiated. Meanwhile, his anemia worsened, with hemoglobin dropping to 5.3 g/dL, and platelet count to 86 × 10^9^/L, without active bleeding. He was transfused with two units of packed red blood cells. His wrist drop deteriorated significantly. Moreover, the presence of thrombocytopenia, the absence of C8 nerve root compression on MRI, and EMG findings showing predominant involvement of C8‐innervated muscles prompted a reassessment of the case, leading to a more detailed occupational history. His family doctor, being aware of his work in a car workshop (Figure 3), suggested screening for lead intoxication. It was found that the patient's blood lead level was significantly elevated (795.2 μg/dL) (0.00–20 μg/dL). Based on these findings, a diagnosis of lead poisoning was established, and chelation therapy was considered.

**FIGURE 2:**
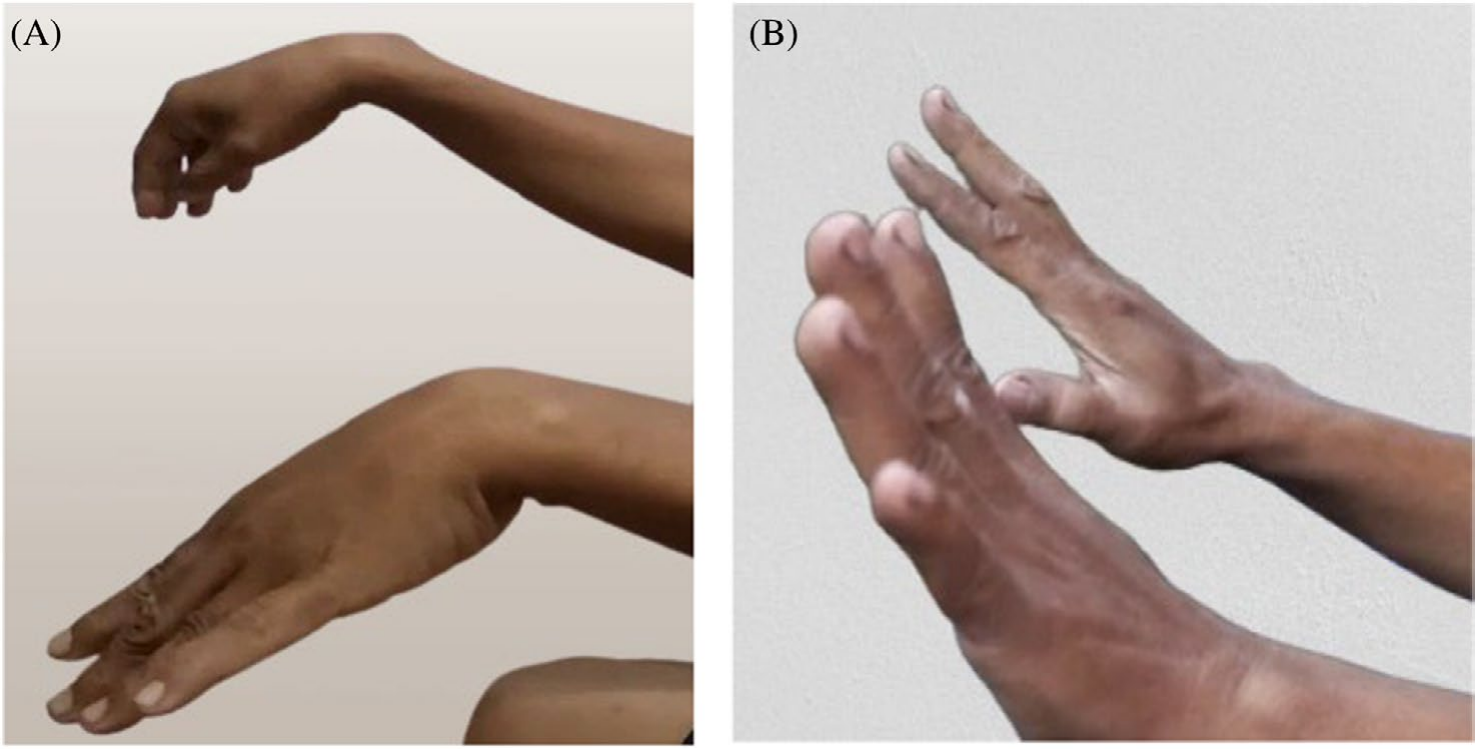
Clinical photographs showing bilateral wrist and finger drop, more severe on the right side (A, before treatment). Marked improvement in wrist and finger extension is observed following treatment (B, after treatment, at 7‐month follow‐up).

Since calcium disodium EDTA was not available locally, the patient was referred to the hospital in Bangkok where 1600 mg/day of calcium disodium EDTA was administered for 5 days. As blood lead level remained high after 4 months, another three‐day course of calcium disodium EDTA at 1600 mg/day was given. His weakness significantly improved after treatment, eventually regaining full strength (Figure 2), with normalization of blood lead level on follow‐up. Blood parameters, including hemoglobin and platelet counts, also returned to normal.

The final diagnosis was chronic motor axonal polyneuropathy due to chronic lead toxicity, with incidental findings of cervical spondylotic radiculopathy and Hemoglobin E disease.

**FIGURE 3:**
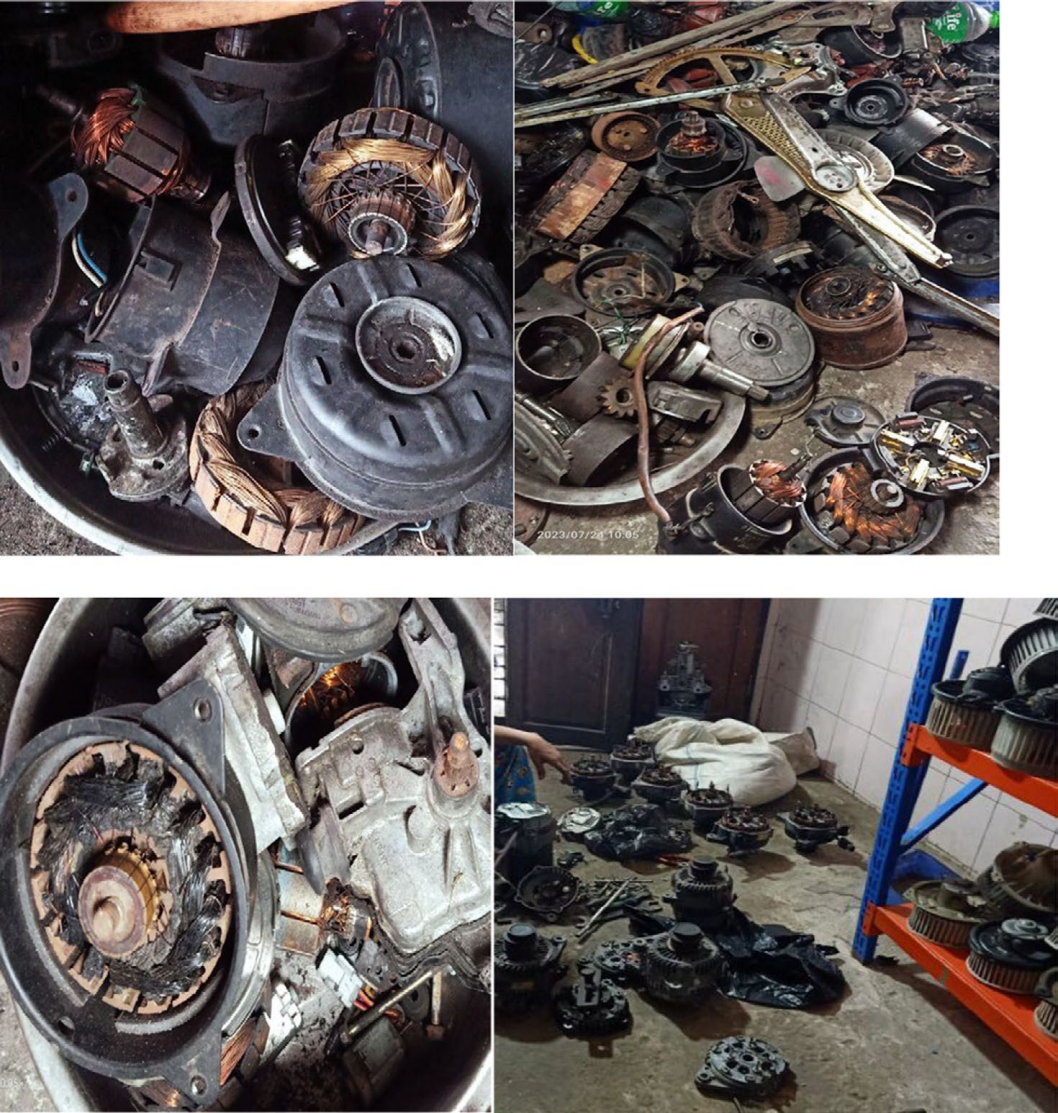
The patient's workplace, a car workshop where old air conditioning units—particularly dynamos—were stored for repurposing.

Upon further enquiry, it was discovered that he repurposed and recycled old air conditioning units (dynamo and other components) imported from other countries in his car workshop (Figure 3). He spent extensive time in his workshop, where particles from these old air conditioning units contaminated with lead were exposed. His family (wife and son), living next to the car workshop within the same compound, also had raised blood lead levels. Although both were asymptomatic, his son—who assisted in the workshop—had a markedly elevated blood lead level that required chelation therapy.

**FIGURE 4:**
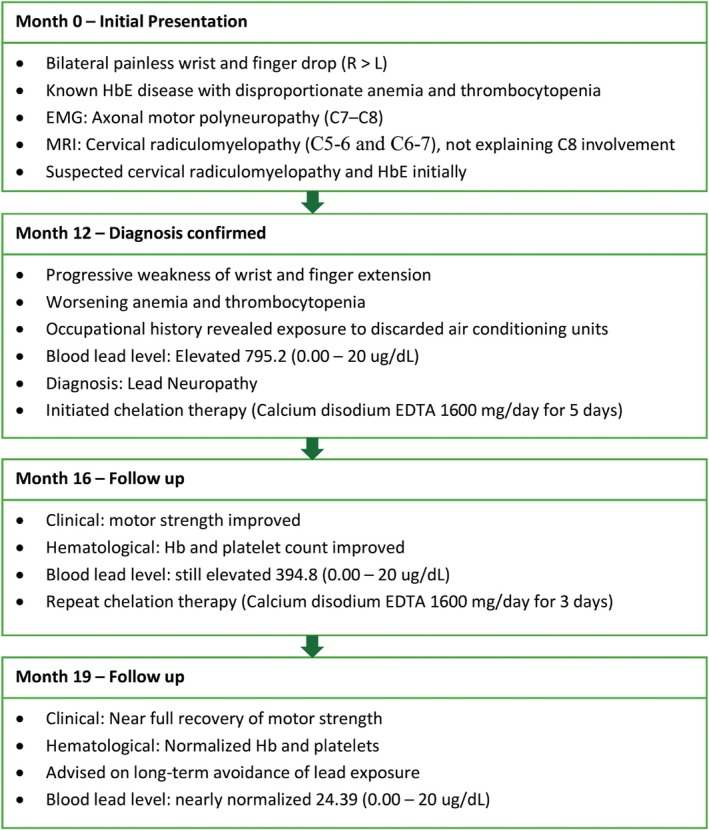
Timeline flowchart summarizing key clinical events, investigations, diagnosis, and follow‐up outcomes in a patient with lead neuropathy over a 7‐month period.

We advised the family to minimize lead dust exposure by improving ventilation in the work area, washing hands thoroughly after handling potentially contaminated materials, using protective measures such as protective clothing, gloves, and masks. Proper disposal of contaminated items was also recommended. A detailed timeline flowchart summarizing the patient’s clinical course, investigations, diagnosis, and follow‐up outcomes over 7 months is presented in Figure 4.

## 3 Discussion

The initial diagnosis of Hemoglobin E disease and cervical spondylotic radiculomyelopathy was based on pallor and limb weakness together with laboratory findings of hypochromic microcytic anemia, hemoglobin electrophoresis of HbE disease, and MRI revealing cervical radiculomyelopathy. However, these did not fully explain the coexistent thrombocytopenia and progressive motor neuropathy. The discovery of occupational lead exposure with significantly high blood lead levels provided a unifying diagnosis—lead neuropathy—which can cause both anemia (via disrupted heme synthesis) and motor‐predominant neuropathy. This was further confirmed by clinical recovery after lead chelation therapy. This case highlights the need for having a high index of suspicion for lead toxicity, particularly in high‐risk exposure settings.

It has long been recognized that lead poses a major threat to both environmental and occupational safety. Lead exposure can damage multiple systems of the body, including the central and peripheral nervous systems, hematology, gastrointestinal system, cardiovascular system, and cause renal impairment, immunotoxicity, and toxicity to reproductive organs. The clinical manifestations affecting the nervous system are reduced attention span, intellectual disability, behavioral disorders, seizures, and neuropathy [5].

Lead can be exposed to the body by inhalation of lead particles as well as ingestion of contaminated food, drink, and dust from the workplace and environment. Lead is mostly used in storage batteries; industries for ammunition, as shielding against radiation; and in solders, paints, and cosmetics.

Lead was commonly used previously in air conditioning units such as in solder joints and electrical components, bearings and lubricants, paints and coatings on metal parts and insulation and wiring, which over time deteriorates and turns into dust that contains lead. Later, restrictions were placed on lead solder due to health concerns [6, 7].

Although lead was phased out globally in large‐scale economies such as stopping the use of leaded petrol and lead paints, small‐scale industries still exist. Furthermore, electronic waste, containing hazardous levels of lead, poses significant environmental and health risks due to improper disposal and informal recycling practices. These unregulated activities contribute to toxic contamination of land, water, and air [3, 8, 9]. Despite effective restrictions in developed countries, many developing countries struggle to prevent lead exposure due to financial constraints and necessity for cost containment [10]. In our case, lead exposure continues as our patient had used discarded, outdated air conditioning units discarded from other countries in his car workshop to save money.

Lead toxicity causes hematologic abnormalities by inhibiting key enzymes in the heme synthesis pathway, such as delta‐aminolevulinic acid dehydratase and ferrochelatase. This leads to ineffective erythropoiesis, accumulation of abnormal heme precursors, and anemia characterized by basophilic stippling and possible hemolysis. Moreover, lead exposure can suppress bone marrow function, leading to thrombocytopenia [11]. In contrast, Hemoglobin E disease is a genetic hemoglobinopathy that causes a mild, stable hypochromic microcytic anemia without associated thrombocytopenia [12, 13]. Thus, the patient's progressive anemia accompanied by thrombocytopenia suggested an additional toxic marrow insult consistent with lead poisoning. Chronic lead toxicity can also cause a reduction in nerve conduction velocity, leading to motor and sensory neuropathies when blood lead concentration is more than 50 μg/dL [5]. Thus, this patient with an excessive increase in blood lead level suffered significant motor axonal polyneuropathy with wrist drop, which resolved after chelation therapy. His anemia and thrombocytopenia were also related to lead toxicity since these were normalized after chelation. Blood lead levels below 5 μg/dL are considered within the normal reference range for adults, although the laboratory we sent gives a normal range of 0–20 μg/dL. Chelation therapy is typically indicated when blood lead levels exceed 50 μg/dL, especially in the presence of clinical symptoms [5, 14].

Lead exposure continues to be an important environmental health problem in developing countries, and clinicians in developing countries should have a high index of suspicion for it. In addition, this case highlighted the importance of occupational history pertaining to the differential diagnosis.

As lead poisoning is a preventable condition, it is essential to implement public health education to increase awareness among the general population. In addition, access to diagnostic testing and chelating agents should be available locally. In the present case, although blood lead level can be locally tested, the patient had to travel overseas to receive chelating therapy, highlighting a critical gap in therapeutic availability. Moreover, healthcare providers, including clinicians, general practitioners, family doctors, nurses, and community health workers, should be adequately informed about the sources, routes, and risk factors of lead exposure to ensure early recognition and prompt management of affected individuals. Given the significant health impacts of lead exposure, all countries, including developing countries, should strengthen their efforts to eliminate the use of lead in order to prevent diseases and disabilities caused by lead exposure.

## 4 Conclusion

This case, initially misdiagnosed as pure Hb E disease and spondylotic cervical radiculomyelopathy, was ultimately identified as lead neuropathy. The diagnosis was aided by the clinical suspicion of the family doctor, whose awareness of the patient's occupational exposure played a crucial role in guiding further evaluation.

## Author Contributions

**Su Mon Thwe:** data curation, formal analysis, investigation, methodology, project administration, writing – original draft. **Htoo Myat Myat Aung:** data curation, resources, writing – review and editing. **Zin Nwe Win:** formal analysis, writing – review and editing. **Ohnmar:** conceptualization, project administration, supervision, validation, visualization.

## Consent

Written informed consent was obtained from the patient.

## Conflicts of Interest

The authors declare no conflicts of interest.

## Data Availability

Data available on request due to privacy/ethical restrictions.
